# Irreconcilable differences?: epistemic injustice and the integration of mental healthcare for LGBTQ+ young people

**DOI:** 10.3389/fpsyt.2026.1868755

**Published:** 2026-09-22

**Authors:** Rachael Eastham, Elizabeth McDermott

**Affiliations:** 1 Division of Health Research, Lancaster University, Lancaster, United Kingdom; 2 School of Social Policy and Society, University of Birmingham, Birmingham, United Kingdom

**Keywords:** epistemic injustice, inequalities, LGBTQ+, mental health, young people

## Abstract

**Background:**

Integrated youth mental healthcare is an approach that has evolved in response to the global escalation of young people’s poor mental health, and the failure of current mental health systems and models to improve this situation. Lesbian, gay, bisexual, transgender, and queer/questioning (LGBTQ+) young people have disproportionately high rates of poor mental health compared with their cisgender, heterosexual peers, but there is a dearth of research examining how best to integrate mental healthcare for LGBTQ+ youth.

**Aim:**

To identify differences in the discursive construction of LGBTQ+ identity, youth, and mental health between LGBTQ+-specific youth mental health services and mainstream services and how these differences may facilitate or limit the integration of mental healthcare.

**Methods:**

We conducted a qualitative secondary analysis using Foucauldian discourse analysis, a method that centralises power and knowledge, to examine the discourses used to construct LGBTQ+ identity, youth, and mental health across four different mental health services. Using epistemic injustice as a theoretical framework, we analysed interviews with LGBTQ+ young people and staff (n = 30).

**Results:**

Mainstream discourses of LGBTQ+ youth mental health concentrate on risks, trauma, and harm, and reinforce a passive, pathologised, and patronised subject position for LGBTQ+ youth. In contrast, we identified three counter-discourses in LGBTQ+-specific mental health services: Knowing and telling, Resisting pathology, and Capability. These counter-discourses allowed for wider, transformative possibilities for LGBTQ+ youth subjectivities and mental wellbeing.

**Discussion:**

Existing models of integrated youth mental healthcare privilege a clinical, developmental, and universal approach that does not consider justice or the oppressive power systems implicated in LGBTQ+ young people’s mental healthcare. Without addressing in/justice, there will remain fundamental irreconcilable differences that will forfeit effective and inclusive systems of mental healthcare for LGBTQ+ young people.

## 1 Introduction

Youth mental health is a global priority. The World Health Organization (WHO) estimates that 1 in 7 young people aged 10–19 experience mental ill health such as depression and anxiety and that suicide is the third leading cause of death for young people aged 15–29 (1). In the UK, estimates are higher, with 1 in 5 young people aged 8–25 in England likely experiencing a mental health condition (2). Despite this concerning prevalence, young people do not have access to timely care that is appropriately designed for them, and when they access services, their experiences are often unsatisfactory and ineffective (3, 4). This is partly because mental health service models and systems have not been designed to identify mental health difficulties early and offer potentially preventive interventions.

Integrated youth mental healthcare is an approach that offers a model of support to improve the mental wellbeing of young people at an early stage. Integration is widely recommended to address the fragmentation of healthcare and involves joined-up, patient-centred services, often delivered through a single community-based site, that attend to multiple health and social needs (5). In contrast, non-integrated approaches to mental healthcare mean specialists and care providers work in disciplinary siloes and are not coordinated in their efforts to deliver support. The provision of integrated youth mental healthcare has been adopted in some high-income countries, such as Australia and the UK, over the past two decades in an effort to improve mental healthcare for young people (3). In a systematic review and meta-analysis, McHugh et al. (4) demonstrated that integrated models for youth mental health produced a small but significant increase in effectiveness for depressive symptoms relative to treatment as usual. They also showed that where studies reported access and engagement (n = 7) effectiveness improved.

Research examining integrated youth mental healthcare suggests that operational issues such as commissioning, workforce, professional work practices, and financial uncertainty challenge the effectiveness of the model (3, 4). Furthermore, the evidence base lacks an examination of the conceptual and paradigmatic foundations of youth mental healthcare that may limit or facilitate integration. In the WHO Mental Health Action Plan (2023–2030), it is asserted that mental health services for children and adolescents must be evidence-based, culturally appropriate, and human rights-orientated (6). This is particularly important for youth who are marginalised and discriminated against based on their age, race, culture, sexual orientation, gender diversity, socioeconomic status, disability, and mental health. The discussions regarding the development of integrated youth mental healthcare have not, so far, considered the implications of thinking about youth mental health inequalities. These models of integrated youth mental healthcare adopt a clinical, developmental, and universal approach (3, 4, 7, 8). For example, McGorry et al. (3: 61), in their discussion of integrated youth mental healthcare, state that ‘the mental health needs of young people are largely universal’. Our concern is that a ‘universal’ approach homogenises need, ignores equality and in/justice, and will not generate effective and inclusive systems of care, meaning those most likely to develop a mental health problem will remain sidelined and excluded.

In our study, the aim was to identify the discourses and underlying theoretical assumptions about lesbian, gay, bisexual, transgender, and queer/questioning (LGBTQ+) identity, youth, and mental health present across different LGBTQ+ youth mental health support services and to examine how these may facilitate or limit the integration of youth mental healthcare. Discourses are important because they have a role in wider social processes and systems of power, which, in turn, may impact what is possible between individuals and services within mental healthcare systems. In the subsequent sections, we outline the evidence on LGBTQ+ young people’s mental health and their service use and experiences. We then describe the rationale for using epistemic injustice as a theoretical approach for the study.

## 2 Background

There is now a substantial body of international evidence that demonstrates elevated rates of poor mental health outcomes for LGBTQ+ young people. Systematic reviews consistently report that sexual- and gender-diverse youth have a higher risk of suicide attempts, self-harm, and depression compared with cisgender heterosexual young people. di Giacomo et al.’s (9) meta-analysis of the estimated risk of suicide attempts showed that trans youth were six times, bisexual youth five times, and lesbian and gay youth four times more likely than cisgender heterosexual peers to attempt suicide. In a 2016 pooled analysis of 12 UK population surveys, people under the age of 35 who identified as lesbian, gay, or bisexual were twice as likely to report symptoms of poor mental health as heterosexual peers (10). In a recent analysis of long-term trends in sexual minority mental health disparities, these disparities had widened (11).

The dominant explanation for this disparity in prevalence is minority stress theory (12), in which unique experiences of LGBTQ+-based discrimination, victimisation, hostility, stigma, and marginalisation lead to increased stress levels and poor mental health. Systematic reviews demonstrate the strong association between experiences of gender- and sexuality-based victimisation and an increased risk of suicide attempts, self-harm, and depression in LGBTQ+ youth (13–16). In a UK survey of LGBTQ+ youth (n = 9,666), the most important ranked predictors of suicide attempts, suicidal thoughts, depression, and anxiety were experiences of LGBTQ+ victimisation, food insecurity, conversion therapy, and having a trusted adult in their lives (17).

Given the higher prevalence of poor mental health amongst LGBTQ+ youth and the ‘minority stress’ that likely causes this burden, it is imperative that mental health systems and service models provide inclusive, effective care. Despite this need, LGBTQ+ young people underutilise mental health services when they need them. In a recent large UK survey on LGBTQ+ youth mental health, 23% of participants did not ask for help, and a further 6% asked for help but did not receive it (17). Hesitancy to access mental health services may be due to fears of discrimination when seeking help, concerns about confidentiality and being ‘outed’, a lack of LGBTQ+ knowledge in services, fears of parents finding out about their LGBTQ+ status, and poor relationships with parents who are required to provide consent or facilitate service access (18–20). Furthermore, evidence reveals that access is made more difficult by multiple marginalisation, especially for trans and non-binary young people and/or those ethnically and culturally minoritised (21, 22).

We argue elsewhere that to improve access to and engagement with mental health services for LGBTQ+ young people, we must address interlocking oppressive systems of power, such as racism and cisheteronormativity, that make getting help difficult (23). For mental health services to work, we suggest an ‘intersectional youth rights approach’ that would address the multiple forms of marginalisation and stigmatisation that LGBTQ+ youth may experience, enable informed, independent decision-making, and uphold the right to freedom of safe self-expression (24). Despite an extensive evidence base demonstrating need and mental health inequities, current debates on integrated youth mental healthcare models and systems of delivery still do not adequately consider justice and the oppressive power systems implicated in LGBTQ+ young people’s mental healthcare. In this study, our aim was to apply social justice thinking to integrated youth mental healthcare for LGBTQ+ young people. In the section below, we outline our theoretical approach to the study using epistemic injustice.

### 2.1 Epistemic injustice

Epistemic injustice is a concept that highlights how knowledge and knowledge-making are key mechanisms in the perpetuation of social injustice. The concept has been used to emphasise how social injustice involves subjugating the knowledges of those on the margins so that they may be undermined as knowers and excluded from the ways in which social meanings are generated within a society. Epistemic injustice was the term coined by Fricker (25), but feminist activists and academics of colour such as bell hooks, Audre Lorde, and Patricia Hill Collins provided the intersectional foundation for thinking about epistemic injustice. In Fricker’s (25) formulation, epistemic injustice takes two forms: testimonial injustice, “when prejudice causes a hearer to give a deflated level of credibility to a speaker’s word” (p. 1), and hermeneutical injustice, where “a gap in collective interpretative resources unfairly disadvantages when it comes to making sense of their social experiences” (p. 1). Here, Fricker is concerned with the everyday epistemic practices of conveying knowledge to others by telling them, such as having to come out as LGBTQ+ or explain to an adult that you are mentally distressed; and through making sense of social experiences, such as being gender diverse or being diagnosed with a mental health condition.

Queer epistemology has a central concern with epistemic injustice and the dominant presumption that sexuality and gender are binary, fixed, stable, and part of innate human nature. As Hall rightly states, the binary conceptions of sexuality and gender are ‘woefully inadequate for knowing the complex experiences and realities of gender and desire’ (26: p. 159). For LGBTQ+ young people, despite gains in LGBTQ+ rights and legislation in some countries, the dominant cisheteronormative discourses of sexuality and gender (where gender identities and bodies align and everyone is assumed to be heterosexual) continue to marginalise and stigmatise LGBTQ+ people and to discriminate against them; consequently, ‘coming out’ takes place on a ‘fraught epistemic terrain’ (26: p. 158). This means that LGBTQ+ young people’s lived experiences of knowing their sexuality and gender may be silenced, undermined, or denied. In Fricker’s (25) terms, this is testimonial injustice, in which LGBTQ+ youth experience an unjust deficit of credibility (27).

LGBTQ+ young people also experience hermeneutical injustice, in which they may not encounter ways of making sense of their own sexuality and gender as a credible, valuable, and legitimate identity. Trans scholarship has highlighted the ways in which gender diversity is shaped by multiple forms of epistemic injustice and epistemological violence that ‘coalesce to delegitimate and extinguish knowledges of gender diversity that do not align with the dominant epistemic and social order’ (28: p. 614). Trans epistemology has utilised the concept of epistemic injustice to address the anti-trans and white supremacist rhetoric that makes trans lives unintelligible, or not knowable, within the dominant prevailing cisheteronormative society (29, 30). Queer education scholars have shown the importance of generating hermeneutic resources to address epistemic injustice for LGBTQ+ young people (31, 32), for example, from schools having a LGBTQ+ inclusive curriculum to providing affirmative knowledge about gender and sexual diversity.

Epistemic injustice as a concept has been deployed within the mental health field for some time. The mental health/disability user movement has been key in exposing the dominant psychiatric knowledge that discredits those with mental health problems (33, 34). Youth mental health and epistemic injustice are less often discussed. A small number of scholars have drawn attention to the ways in which children and young people’s knowledge is not taken seriously by adults and, as a consequence, they are easily discredited. Adult knowledge is consistently positioned as more credible and is privileged in ways of knowing (35, 36). This means that young people are often treated as objects to be protected and controlled because it is assumed they do not know what is best for themselves, hence rendering them passive and unheard within epistemic regimes. LeFrançois (36) argues that children and young people with mental health difficulties are subject to twofold epistemic injustice through being discredited as young and mad. Arguably, LGBTQ+ young people with mental health problems are discredited because they are young, mad, and queer (37, 38).

For this analysis, we used the theoretical frame of epistemic injustice because we were concerned with the role of discourse and knowledge in social processes of legitimation and power.

The aim of this study was to identify how the concepts of LGBTQ+ identity, youth, and mental health were discursively constructed across LGBTQ+-specific youth mental health services and then to compare these constructions with those in mainstream youth mental health services. Within this, we wanted to examine how the different discourses of LGBTQ+ identity, youth, and mental health circulating within mental health services might be implicated in epistemic in/justice and how any differences may influence collaboration across health and social care services required for effective, inclusive, integrated youth mental healthcare. In this paper, we address the study’s two main research questions through this lens: 1) What discourses are used to construct LGBTQ+ identity, youth, and mental health across different mental health services? 2) How do different discursive constructions of LGBTQ+ identity, youth, and mental health limit or facilitate integration of care?

## 3 Materials and methods

### 3.1 Rationale for using Foucauldian discourse analysis

Our project used Foucauldian discourse analysis (FDA) to interrogate secondary qualitative data from the Queer Futures 2 study (www.queerfutures2.co.uk). These data were collected in 2020–2021 from 12 diverse case study sites that provided LGBTQ+ youth mental health support, including National Health Service (NHS), voluntary sector, local authority, and education services. The data showed conflicting ways of understanding LGBTQ+ identity, youth, and mental health across different types of services. For example, NHS services tended to consider specific mental health provision for LGBTQ+ youth unnecessary because statutory support, i.e., public services provided by government and local authorities, is nominally equal and ‘open to all’ (39). This contrasted with voluntary organisations that provided LGBTQ+-specific mental health support that focussed on the importance of affirming LGBTQ+ identity.

In FDA, language and discourse are understood as social practices through which meaning, knowledge, and beliefs are produced. This methodology asks questions about the relationship between discourse and how people think or feel (subjectivity), what they may do (practices), and the material conditions within which such experiences may take place (40). FDA examines the availability of discursive resources within a society (i.e., a discursive economy) and the implications of this for those who live within it. In addition, FDA is concerned with the role of discourse in wider social processes of legitimation and power. Since discourses make available ways of seeing and being, they are strongly implicated in the exercise of power. Dominant discourses privilege those versions of social reality that legitimate existing power relations and social structures. For example, expert psychiatric discourse, which is embedded in statutory mental health services, defines mental health as pathological. Some discourses are so embedded that they become ‘taken for granted’ and very difficult to challenge, such as the discourse that young people need protection rather than autonomy.

FDA was chosen as an analytical strategy because it specifically attends to the role of discourse in knowledge and power. In our project, FDA was based on critical psychological research that draws on poststructuralist understandings of how discourses operate (40, 41). FDA has previously been used successfully to generate in-depth understandings of the mental health of LGBTQ+ young people (38, 42).

### 3.2 Foucauldian discourse analysis method

There is no set procedure for Foucauldian discourse analysis (FDA) (40, 43). Instead, FDA is characterised by a ‘top down approach to interpretation’ (44, p. 279), whereby analysis starts with conceptual tools derived from theory—in this case, conceptualisations associated with LGBTQ+ identities, youth, and mental health.

#### 3.2.1 Data selection

Our primary Queer Futures 2 (QF2) dataset included interview transcripts collected in 2020–2021 from staff and LGBTQ+ young people (N = 86) at 12 LGBTQ+ youth mental health sites. See McDermott et al. (24) for more details. Arribas-Ayllon et al. (43) suggest a range of criteria for the selection of a sample of texts for discourse analysis. In this study, we were interested in 1) texts that show how discursive objects are described and explained (LGBTQ identity, youth, and mental health); 2) texts that construct a social problem (integrated care); and 3) descriptions of practice that illustrate how an object is acted upon, i.e., how objects are regulated (LGBTQ+ youth navigation of mental health services). Using these criteria, we selected four of the 12 case study sites. We included case study data from a Trans+-specific youth service to ensure that we attended to gender diversity. **Table 1** provides details of the data (N = 30 texts) included in this project across the selected case study sites.

**Table 1:** provides details of the data (N = 30 texts) included in this project across the selected case study sites.

Site label	Service type	Location	Number of texts (interviews)
CS4	Integrated	North-West England	N = 7 (4 staff; 3 young people)
CS5	LGBTQ+ youth	South-East England	N = 9 (3 staff; 6 young people)
CS10	Integrated	South-West England	N = 6 (3 staff; 3 young people)
CS12	Integrated	North-West England	N = 8 (5 staff; 3 young people)

#### 3.2.2 Data analysis

We used Willig’s six stages of FDA (40) (see **Table 2**) to analyse 30 texts from our four selected case study sites. Willig’s six stages of Foucauldian discourse analysis Cognisant of how the steps for FDA provide ‘ways of approaching texts rather than recipes for producing correct analyses’ (40: p. 128), we conducted FDA across all texts to identify discursive constructs for each of the three objects—LGBTQ+ identities, youth, and mental health. Having completed FDA for all the texts, we identified 208 discursive constructions that occurred more than once. From these 208, we selected the discursive constructions that were most relevant to our research questions using the following criteria: 1) the frequency of the discursive construction across the texts; 2) its conceptual relevance to the research questions; and 3) its conceptual relevance to epistemic justice. This produced the 27 discursive constructions most relevant to our discursive objects: LGBTQ+ identity (n = 10), mental health (n = 12), and youth (n = 5). We then consolidated these 27 most relevant discursive constructions into eight overarching ‘key discursive constructions’ by clustering related discursive constructions (see **Table 3**).

**Table 2:** Willig’s six stages of Foucauldian discourse analysis.

Stage	Analytic activity
Discursive constructions	Identifying how the discursive objects (LGBTQ+ identity, youth, and mental health) are constructed in the texts.
Discourses	The focus here was on differences between these constructions and their interrelationship (e.g., biological vs. social constructions of LGBTQ+ identity).
Action orientation	Examination of the discursive contexts within which different constructions of the object are being deployed. What is the function of constructing the object in this way?
Positionings	Having identified different constructions of the discursive object in the text, and located them within wider discourses, the subject positions they make available become the analytical focus.
Practice	Consideration of what can and cannot be said and done from these subject positions. A systematic exploration of the opportunities for action in practice.
Subjectivity	Attend to the relationship between discourse and subjectivity, i.e., what can be felt, thought, and experienced from these different subject positions.

**Table 3:** Relationship between the most relevant discursive constructions and key discursive constructions.

Discursive object	Discursive constructions (N = 27)	Key discourse (N = 8)
LGBTQ+ identity	Constructed as risk of judgment	Minoritisation stress
	Constructed as risk/trauma	
	Constructed as work	
	Constructed as belonging	(Whose) community
	Constructed as Queer, Trans and Intersex Black people and People of Colour (QTIBPOC)	
	Constructed as disclosure	Knowing and telling
	Constructed as fluid	
	Constructed as something valid to explore	
	Constructed as pronouns	
	Constructed as labels/terminology	
Youth	Constructed as able to lead	Capability
	Constructed as able to decide	
	Constructed as power imbalance	Power
	Constructed as contingent on parents/carers	
	Constructed as needing support to access/navigate systems	
Mental health	Constructed as involving feelings	Resisting pathology
	Constructed as embodied	
	Constructed as sociality	
	Constructed as communication	
	Constructed as related to wider world/social events	
	Constructed as liberation	Liberation and empowerment
	Constructed as trans-normativity	
	Constructed as empowerment	
	Constructed as confidence	
	Constructed as (trans) youth work	Involves labour
	Constructed as individualised to needs	
	Constructed as work	

Relationship between the most relevant discursive constructions and key discursive constructions Finally, these eight key discursive constructions became our focussed units of analysis framed by epistemic injustice. This final stage examined how these key discourses work and directly answered research question 2: How do different discursive constructions of LGBTQ+ identity, youth, and mental health limit or facilitate integration of care? In this paper, we present three of these key discursive constructions in more detail—knowing and telling, resisting pathology, and capability—because they were most important for considering the research questions.

### 3.3 Ethics

The Queer Futures 2 study received NHS ethical approval [19/NW/0642] and the research team already had direct access to the data. However, we did not automatically assume this meant the project posed no ethical risks. Importantly, we considered whether the secondary data analysis objectives were ‘discordant’ with the original research objectives, i.e., whether consent given by Queer Futures 2 was for an entirely ‘different’ project with incommensurate aims and objectives. We committed to ensuring that the data were handled in a way that did not undermine the original informed consent given by participants, i.e., consent to produce impactful outputs within the original project remit (‘what works to support LGBTQ+ youth mental health’), albeit using a different methodology. There were no immediate risks of harm to the research team, who were familiar with the dataset, and we did not require new ethical approval for this study. We also worked closely with project advisors from two of our original case study sites to validate decisions and steer the process.

## 4 Results

The results that follow are supported by verbatim quotes that use the language of the participants.

### 4.1 LGBTQ+ identity: knowing and telling

Our analysis revealed three discursive constructions comprising ‘knowing and telling’: i) LGBTQ+ identity as disclosure; ii) LGBTQ+ identity as fluid; and iii) LGBTQ+ as labels.

#### 4.1.1 LGBTQ+ identity as disclosure

This discourse highlights an inherent tension in knowing and telling about LGBTQ+ identity. To ‘come out’ functions to demarcate difference, inherently reinforcing cisheterosexuality. This contrasts with normative cisheterosexual identities that are assumed and require no explanation within mental health services. Where disclosure is expected or demanded by ‘others’, such as mental health services or professionals, the imperative to disclose arguably serves the system rather than the individual. ‘Data’ requested by services like that on referral or intake forms mean that LGBTQ+ individuals are knowable, labelable, and countable in the terms (and language) defined by the service (which bolsters constraint and regulation).

However, for individuals, disclosure may be an autonomous act—important for building community, celebrating, and being proud. Disclosure can facilitate unity with others, mobilise change, permit access to specific services, and/or enable ‘being counted’. Nonetheless, the energy involved in navigating cisheteronormative contexts and disclosure can impose a burden on LGBTQ+ youth that impacts their mental health.

Considering the context and knowing that disclosure is not equally available to everyone or in all contexts, we gain deeper insight into burdens on LGBTQ+ youth mental health. In all cases, doing disclosure requires resource. Emotional resource, as an individual navigates fear, uncertainty, risk; or at the very least, the ongoing burden of disclosure, as highlighted in the quote:

CS12 Staff Member: “I think some of the challenges are the fact that LGBT young people have to come out and the stresses that is involved in that process. It’s an ever ongoing process whether it’s with their family, whether it’s with their friends, whether it’s in school, whether it’s in college, and different aspects of the family. And it goes on and on and on.”

Hermeneutic resources—the shared interpretive discursive tools, such as concepts, words, and narratives, used to understand experiences, communicate meaning, and make sense of the world—inform how disclosure and the explanation of identities are possible within services: what language is known, what language is available in the wider lexicon, and what is considered ‘valid’ by normative systems. However, hermeneutic injustice—the paucity of ways for LGBTQ+ youth to explain their identities meaningfully—and their lack of credibility through testimonial injustice function to strain LGBTQ+ youth during moments of disclosure.

The need to bolster both young people’s emotional and epistemic resources to navigate the wider world (including mainstream mental healthcare systems) is one reason why ‘coming out support’ is a core offer by specialist LGBTQ+ youth (mental health) support services.

#### 4.1.2 LGBTQ+ identity as fluid

CS10 Staff Member: “I think just a space for people to come where they don’t, they know that there’s not going to be any judgement, basically. And that they can be anyone that they wanna be. And that if next week they come and be someone completely different then that’s totally cool.”

This particular discourse resists cisheteronormative demands to be knowable and labelable. Fluidity and exploration of sexual and gender identity confound individuals, services, and systems in efforts to know and tell in a context where a preoccupation with labelling and counting is a cornerstone of heteronormative regulatory systems. This discourse validates (LGBTQ+) diversity and change, including possibilities for people otherwise known as cisheterosexual, thereby countering the notion that changes in gender/sexual identity indicate uncertainty or represent phases or trends. In many texts in our analysis, understanding LGBTQ+ identity as fluid was why disclosure was constructed as unnecessary. Instead, creating space for exploration without demanding a ‘final destination’ that can be labelled and articulated was preferred. LGBTQ+ diversity and fluidity render LGBTQ+ identities inexplicable in the context of normative systems (mainstream services) that seek to regulate and explain them.

In terms of epistemic justice, resistance occurs through a refusal to participate in the conversation about ‘what LGBTQ+ identities are’ in ways that are intelligible to wider cisheteronormative systems. Conversely, withholding this information arguably depletes the hermeneutic resource that facilitates possibilities for being known and for validating the existence of LGBTQ+ people.

#### 4.1.3 LGBTQ+ identity as labels

CS5 Staff Member: “A lot of our young trans people talk about [how its]the only space where they can just not be trans. They can be someone who’s not identified by their label, which is so ironic that you go to a trans service and then you can be there and not have to be trans, right? It’s, you know, it’s both absolutely creating this unity with being trans and finding power in that, finding joy in that. But then these labels are not ones that we’ve necessarily created ourselves or given to ourselves. A lot of labels can be implemented on us because we are ‘other’, right, and labels like that can be useful because it can help us find other people who are ‘othered’ in the same way. But, you know, you just have to see how a trans person is represented in the media to see how it’s a sensationalist way of putting someone in a box.”

This discourse is grounded in the practice of knowing and telling about LGBTQ+ through pronouns and the use of specific LGBTQ+ language. Discourses that construct LGBTQ+ identity as pronouns and/or labels are reductive and pose risks of reducing understanding of LGBTQ+ identities to dictionary-based definitions only. Using label/s to explain sexual and gender identity can reinforce fixity and, as indicated in the quote, raise questions about the architecture, owners, and function of labels, i.e., about their role in making people knowable and categorised by others. Labels also have less relevance when LGBTQ+ identity is fluid and when they present possibilities for ‘fixing’ people into position as a ‘specific type of person.’ Thus, ‘unlabelling’ can be a powerful form of resistance.

Conversely, proficient knowledge and practices around pronouns and labels can also function as a helpful shorthand for identification and allyship with peers, adults, services, and so on. In the current historical moment (especially) trans youth face specific forms of (epistemic) injustice across a hostile policy and legislative landscape. Therefore, literacy around pronouns serves as an important way to signify trans inclusion.

Overall, the discourse of knowing and telling is heavily mediated by forms of epistemic injustice. Fundamentally, LGBTQ+ youth with mental health challenges are not considered credible knowers and narrators of their own identities and experiences. The fluidity of LGBTQ+ youth identities and the language used to explain them are readily undermined by a normative system that demands fixity and that knowledge be demonstrable (observable and measurable) to be comprehensible in dominant neoliberal cisheterosexual frameworks. Dominant normative systems also expect to be the architects and arbiters of language, in control of hermeneutic resource. Thus, the marginalisation and invalidation of LGBTQ+ language are limiting for anyone else who wishes to meaningfully understand LGBTQ+ youth mental health.

### 4.2 Youth: capability

Through our analysis, we identified a discourse of youth as capability that functions to resist normative discourses of youth that reinforce incapability, lack of understanding, and inferiority compared to adults. Instead, this discourse demonstrates the value of youth as knowers, collaborators, and change makers. Here, capabilities relate to youth being able to know themselves and make decisions about what they need for themselves in relation to gender and sexual identity and mental health. This contrasts with normative discourses that situate LGBTQ+ youth as unable to know their gender and identity (and mental health) thereby perpetuating testimonial injustice. We identified two ways that youth were discursively constructed as capability: i) youth as able to decide; and ii) youth as able to lead.

#### 4.2.1 Youth as able to decide

CS4 LGBTQ+ Young Person: “They didn’t really keep anything from me or make me feel like I wasn’t in control of the decision but if I didn’t know what u needed they would advise me in what they think might be best but still let me chose.”

From our analysis, we identified a discourse of youth as being able to make decisions for themselves (e.g., about their healthcare, gender, and sexual identity) and to do so in collaboration with adults. Although ‘shared decision-making’ is a more established credo in adult (mental) healthcare, it is arguably written out of normative mental health support systems for youth, as they so frequently require consent and permission from adults, e.g., parents, and responses such as safeguarding loom large over youth interactions with adult professionals.

By contrast, the texts within this analysis, as evidenced by the quote above, validate youth decision-making capabilities. This discourse highlights the diversity of young people, their unique needs, and their own knowledge of these needs. An important aspect of this discursive construction, with associated benefits for their mental health, was the emphasis on their feelings of control. ‘Feeling’ in control, rather than being in control, arguably functions more ‘realistically’ because this framing reinforces the importance of autonomy whilst recognising that for youth, until at least 18 years old when they reach adulthood, there are many aspects of their own lives they are not permitted to control. This does not reflect youth capabilities but, instead, reflects systems that regulate us socially.

#### 4.2.2 Youth as able to lead

CS12 Staff Member: “We can’t tell them what’s going to work for them and we also don’t, we can’t put ideas into their head, or say, “this is your trail, this is your pathways, this is what you need to be doing”, like a tick box. It’s not like that. The sessions are: “what is gonna work for you” “what’s gonna, these are your goals, these are your sessions and how are we going to support you, what do you need?”

Typical approaches to youth presume parents/carers, teachers, and older adults to be inherently more capable because of their age. In this discourse, however, youth are identified as capable of leading themselves, other young people, and adults. In a normative frame, being ‘able to lead’ arguably reinforces constructions of youth as ‘the future’ and imperatives for them to build that future effectively (e.g., contributors to the labour market). However, in the context of this discourse, leadership is associated with self-governance, activism, and resistance against regressive and discriminatory contexts regarding gender/sexual identity and youth—thus positioning young people as counter-normative ‘agents of change’ who further LGBTQ+ ideals rather than normative goals.

Where youth can lead, these discourses are explicit and implicit in their characterisation of youth as active. This resists the ways in which youth are normatively positioned as helpless and incapable and, for those reasons, vulnerable and in need of adult direction. Instead, youth capabilities are valued and mobilised under schemes of ‘youth leadership’ by adults to help support decisions about young people’s lives and care and to help design services and activities that are suitable for their needs. So, whilst normative social, political, legal, economic, and health systems typically discredit young people’s knowing, in this discourse, there is an active redress of this injustice through being guided by youth knowledge and preferences.

### 4.3 Mental health: resisting pathology

Our analysis identified a strong dominant discourse that described resistance to the pathologisation of LGBTQ+ young people’s mental health. We identified two discourses that constructed mental health as ‘resisting pathology’: i) mental health as embodied feelings; and ii) mental health as sociality.

#### 4.3.1 Mental health constructed as embodied

CS5 LGBTQ+ Young Person: “We have group conversations about struggles we’re facing and how we deal with those struggles and youth workers are always there to talk and other people are always around to talk. Sometimes just letting it all out with someone who understands what you’re going through can be extremely helpful. Having a safe space to talk about trans specific issues really helped my mental health and I would always look forward to going to CS5 because I always feel better after going.”

As this quotation suggests, this mental health discourse centred on the importance of young people articulating their feelings and of these feelings being heard and acknowledged. Within a trusting environment, young people’s feelings and emotions were not disparaged, denied, or categorised as disordered but were validated. In this discourse, the body features in descriptions of mental health as feelings, e.g., ‘letting it all out’, decompression, relief, escape, relaxation, or a weight lifted. These embodied feelings were especially key in our study to the wellbeing of trans and non-binary young people.

This discourse of mental health as embodied feelings was an explicit rejection of pathologising young people’s emotions and feelings. This is a counter-discourse to mainstream psychiatry, in which feelings and emotions are judged in relation to rationality and as requiring regulation. Within dominant understandings, young people are considered over-emotional and immature because they have not ‘mastered’ or regulated their emotions. In the psychiatric paradigm, emotions/feelings need to be ‘appropriate’, in that they are regulated or are as socially expected in response to life events considered socially important, such as bereavement. If they are not, then they are categorised as psychiatric indicators requiring diagnosis and treatment regimes.

A mental health discourse that constructs feelings and emotions as valid addresses the affective dimension of epistemic injustice and makes possible the reduction of suffering. This emotion-centred construction of mental health de-pathologises, humanises, usualises, and acknowledges the feelings/emotional distress and suffering/mental health experiences of LGBTQ+ young people. This discourse enables the opportunity for trans and queer embodied mental wellbeing.

#### 4.3.2 Mental health constructed as sociality

CS4 Staff Member: “I think because a lot of the heterosexual or cisgender population especially with younger people is under the impression that things are fine, or that there’s no stigma anymore and everything’s cool and groovy, etc etc, that a lot of younger people I’ve worked with tend to chastise themselves when they don’t feel ok, because the message is “everything’s ok”[ … ] So, there’s that huge kind’ve work in validating it and being like: it’s ok not to be ok, there are reasons for this … it’s kind’ve recognising and validating the impact of things like heteronormativity.”

In our analysis, mental health was also discursively constructed as feelings/emotions that were generated by the wider social world. In this quotation, the LGBTQ+ youth practitioner explains how mental health is constructed explicitly in relation to intersecting power and structures, e.g., cis-heteronormativity, white supremacy, and adultism. In this discourse, there was nothing abnormal in feeling distressed by these cis-hetero white supremacist norms that do not welcome your existence or, indeed, deny your right to exist. This acknowledgement of epistemic injustice is counter to dominant notions of mental health as individualised psychopathology, in which disordered brains, genes, or psychologies within the individual are considered the cause of any problems.

Mental health constructed as sociality means that it is understandable—neither irrational nor incomprehensible—that these systems of oppressive power impact the mental health of LGBTQ+ young people. ‘Struggles’ become manageable, valid, and normalised when mental distress is constructed as the affective or psychic cost of not following a normative existence (45, 46). Struggles also result from epistemic injustice, in which LGBTQ+ young people are unable to make their experiences intelligible or to articulate and address their needs.

In this discursive construction of mental health as sociality, good mental health is created through connection and trusted collaboration with others, such as peers, groups, and communities. Mental health constructed discursively as sociality allows for the creation of shared meanings and the creation of hermeneutic resources to navigate the normative world that can deny, discriminate against, and denigrate diverse genders and sexualities.

## 5 Discussion

The aim of this study was to identify how the concepts of LGBTQ+ identity, youth, and mental health were discursively constructed across LGBTQ+-specific youth mental health services and then to compare these with mainstream youth mental health services. Evidence (39) and our own experiences generating data through Queer Futures 2 suggested that there were differences between LGBTQ+ youth specialist and mainstream conceptualisations that may be problematic for the development of effective integrated youth mental healthcare. At the heart of this study was the idea that discourse, knowledge and power were fundamental to understanding why there might be difficulties with existing integrated youth mental healthcare for LGBTQ+ young people. Our supposition was that, without attending to the social injustices that some youth might face, inequitable and insufficient understandings of LGBTQ+ youth mental health will mean that this model of care will not be effective for those most disadvantaged and most likely to develop mental health difficulties.

We used epistemic injustice as a frame of thinking for the study because knowledge and knowledge-making are important mechanisms that perpetuate injustice. The question of whose knowledge is validated and who has epistemic authority and who does not is a question about knowledge and power. The capacity of LGBTQ+ youth with mental health problems to know is questioned by mainstream society. Their LGBTQ+ identity may be constructed as a ‘phase’ or as something they are unsure about, or it may be denied. Their age means they are ‘too young to know’, and their mental health status is one in which they cannot be trusted to know. This discrediting of LGBTQ+ youth as knowers may also occur at the intersections of other oppressive power systems, such as race, social class, and disability.

Our Foucauldian discourse analysis revealed some significant incompatibilities in the ways LGBTQ+ identity, mental health, and youth are known in LGBTQ+-specific mental health support when compared with mainstream discourses. Our analysis of LGBTQ+ identity discourses showed that the dominant discourse of ‘knowing and telling’ sits at a tension point between forms of LGBTQ+ resistance and being beholden to oppressive power systems. What is known about LGBTQ+ identity, by whom, and how (i.e., through what knowledge systems and by whom they were created), and what is then communicated about LGBTQ+ identities and for what reason are crucial here. Fundamentally, LGBTQ+ youth with mental health challenges are not considered credible knowers and narrators of their own identities and experiences. LGBTQ+ youth fluidity of identity and the language to explain identities, are readily undermined by a normative system that demands fixity and categorisation and that knowledge be demonstrable (observable and measurable) to be comprehensible and governable in mainstream mental health services.

We found a similar incongruence across the concept of mental health. Dominant discourses of LGBTQ+ youth mental health concentrate on risks, trauma, and harm. These discourses only make possible a passive, pathologised, and patronised subject position. In our dataset, mental health is constructed as embodied feelings and sociality as represented through the discourse Resisting Pathology. An individual, when experiencing difficult feelings/emotions, is not a failure or disordered. These difficult feelings, if centred via talking and community, can counter normative discourses (and build hermeneutic resources) and generate feelings of relief, affirmation, safety, and joy. Resisting pathology posits the possibility of a mentally well subject position. In contrast, mainstream mental health discourses construct mental health as a problem within the individual, a medical matter, and something that is understandable only through diagnostic (imperialistic) criteria, then ‘treated’ with an accompanying expectation of responsibility and self-management. Psychiatry is the ‘regime of truth’ with regard to mental health. So, ‘resisting pathology’ is possibly a ‘reverse discourse’.

We also found a discordant discourse regarding the construct of youth. The dominant way of understanding youth is inherently epistemically unjust. Epistemic authority requires power and youth power is always limited compared to adults. Testimonial injustice caused by prejudice in the economy of credibility for young people is actively reinforced through, for example, legislation that insists that young people cannot make adult decisions until they are 18 years of age, or that pays younger people less for the same jobs as older adults, or that denies access to gender-affirming healthcare. In the absence of power and credibility, LGBTQ+ youth are typically not afforded the opportunity to decide for themselves. This contrasts with the discursive construction of youth as capability that we found in our dataset. This discourse demonstrates the value of youth as knowers, collaborators, and change makers. Here, capabilities relate to youth being able to know themselves and make decisions about what they need for themselves in relation to gender and sexual identity and mental health. This discourse privileges youth as knowers.

What does this mean for the possibility of integrated youth mental healthcare? Are these incompatibilities across LGBTQ+ youth specialist and mainstream services reconcilable? For example, in terms of LGBTQ+ identity, how can organisations and different parts of the healthcare system collaborate and communicate effectively when mainstream services seek to know through counting and labelling using predefined ‘boxes’, whilst LGBTQ+ lives are, in reality, diverse, fluid, and therefore inexplicable in the context of these normative systems. Knowing and telling is arguably a basic foundation for any service to be able to offer effective support to any service users or collaborate with partner organisations. However, when the system is set up in rigid ways that seek to regulate and explain LGBTQ+ identities ‘top down,’ this forfeits meaningful space within which LGBTQ+ youth can be understood and their needs can be responded to.

If these differences are to be reconciled, an LGBTQ+-inclusive and successful integrated youth mental healthcare model has to move away from a clinical and diagnostic approach to a more explicit non-pathologising support. This integrated model needs to think less about developmental age norms and more about enacting young people’s human rights that emphasise their involvement and say in their care. Finally, if an integrated model of youth mental healthcare is to be effective, it must recognise that LGBTQ+ young people are subject to many injustices and address these barriers through LGBTQ+-affirming care and support. The findings from this study suggest that we must address epistemic injustice, in which young people are discredited as knowers with regard to their sexuality, gender, and mental health, in integrated youth mental healthcare models; otherwise, this approach will be exclusionary and ineffective for LGBTQ+ young people.

The findings are limited by an FDA approach that is necessarily labour-intensive, small-scale, and unable to make generalisations. In addition, handling the analysis in a way that was structured around predefined discursive objects—LGBTQ+, youth, and mental health—and arranging the analysis in this way may imply a (false) distinction between certain discursive constructions. However, this small-scale and theoretically rich study has provoked thinking about how to deliver effective, inclusive integrated youth mental healthcare to LGBTQ+ young people.

## Data Availability

The data is restricted because as qualitative datasets they posed risks for being identifiable for LGBTQ+ young people including those aged under 18. Requests to access these datasets should be directed to r.eastham1@lancaster.ac.uk.
